# Insistence on sameness relates to increased covariance of gray matter structure in autism spectrum disorder

**DOI:** 10.1186/s13229-015-0047-7

**Published:** 2015-10-01

**Authors:** Ian W. Eisenberg, Gregory L. Wallace, Lauren Kenworthy, Stephen J. Gotts, Alex Martin

**Affiliations:** Laboratory of Brain and Cognition, National Institute of Mental Health, Bethesda, MD USA; Department of Speech and Hearing Sciences, The George Washington University, Washington, DC USA; Center for Autism Spectrum Disorders, Children’s National Medical Center, Rockville, MD USA

**Keywords:** Autism, Insistence on sameness, Repetitive behavior, MRI, Structural covariance, Subcortex

## Abstract

**Background:**

Autism spectrum disorder (ASD) is characterized by atypical development of cortical and subcortical gray matter volume. Subcortical structural changes have been associated with restricted and repetitive behavior (RRB), a core component of ASD. Behavioral studies have identified insistence on sameness (IS) as a separable RRB dimension prominent in high-functioning ASD, though no simple brain-behavior relationship has emerged. Structural covariance, a measure of morphological coupling among brain regions using magnetic resonance imaging (MRI), has proven an informative measure of anatomical relationships in typical development and neurodevelopmental disorders. In this study, we use this measure to characterize the relationship between brain structure and IS.

**Methods:**

We quantified the structural covariance of cortical and subcortical gray matter volume in 55 individuals with high-functioning ASD using 3T MRI. We then related these structural metrics to individual IS scores, as assessed by the Repetitive Behavior Scale-Revised (RBS-R).

**Results:**

We found that increased coupling among subcortical regions and between subcortical and cortical regions related to greater IS symptom severity. Most pronounced, the striatum and amygdala participated in a plurality of identified relationships, indicating a central role for these structures in IS symptomatology. These structural associations were specific to IS and did not relate to any of the other RRB subcomponents measured by the RBS-R.

**Conclusions:**

This study indicates that behavioral dimensions in ASD can relate to the coordination of development across multiple brain regions, which might be otherwise obscured using typical brain-behavior correlations. It also expands the structures traditionally related to RRB in ASD and provides neuroanatomical evidence supportive of IS as a separate RRB dimension.

**Trial registration:**

ClinicalTrials.gov NCT01031407

**Electronic supplementary material:**

The online version of this article (doi:10.1186/s13229-015-0047-7) contains supplementary material, which is available to authorized users.

## Background

Autism spectrum disorder (ASD) is a neurodevelopmental disorder characterized by social-communication deficits as well as repetitive behaviors and narrow, restricted interests (restricted and repetitive behavior (RRB); [2]). The latter is a particularly heterogeneous domain, encompassing a wide range of symptoms including stereotyped and repetitive behaviors, unusual sensory sensitivity, ritualistic behavior, and insistence on sameness. Initial attempts to explain all of ASD symptomatology in terms of broad neurocognitive principles have given way to pursuing separate, potentially independent correlates of these two core symptom domains [24]. However, recent work behaviorally characterizing the RRB domain implies that increased granularity is needed to adequately pursue the neuroanatomical underpinnings of ASD symptomatology.

Specifically, factor-analytic studies characterizing the nature of the RRB domain consistently find that RRB breaks down into two or three subdomains, often separating repetitive sensory and motor behaviors from insistence on sameness (IS; [9, 34, 39, 42, 49, 58]). IS severity has been found to be relatively independent of IQ and age [7, 30] in contrast to repetitive sensory and motor behaviors, which is more common among those with lower IQ levels and younger ages [50]. IS is therefore the component of RRB consistently found across the entire spectrum of functioning levels and age ranges and is a prominent RRB deficit in higher functioning and older individuals with ASD. There is also evidence of familial aggregation for IS using sibling-pair correlations [9, 34, 55, 58], suggesting a specific genetic underpinning. IS could be conceptualized as a separable dimension of RRB in ASD, especially prominent in high-functioning ASD, given prior factor-analytic studies and its relative independence from IQ and age.

In addition to behavioral atypicalities, ASD is also characterized by widespread neuroanatomical, including subcortical, abnormalities. Subcortical abnormalities in ASD affect many structures. For example, studies have shown increased volume of the basal ganglia and thalamus [17, 59], and the caudate [26, 28, 37, 52] in ASD compared to typically developing (TD) individuals. Other work has also shown that the developmental trajectories of the striatum, particularly the caudate, during adolescence and early adulthood are affected in ASD [38]: caudate volume increased in ASD, while it decreased over time in TD controls. This work was verified in a longitudinal study [35], which found that the growth rate of the caudate was increased in adolescents with ASD versus controls. Differences in other structures have also been observed in ASD, including enlarged hippocampal volume [4, 52, 54] and abnormal amygdala growth [53, 54, 57], though directionality is inconsistent.

While increased striatal volume in ASD relative to TD groups is relatively well established (although see Gaffney et al. [22]; Herbert et al. [27]), the relationship between subcortical structural volume and RRB remains contentious [36]. Some authors have found a positive relationship between caudate volume and RRB [28, 35, 52], while others find the opposite [17, 38]. Additionally, the amygdala has been implicated in RRB, particularly ritualistic behavior [16], extending the range of candidate subcortical structures related to RRB. These inconsistent findings may be rooted in a variety of factors. ASD is an inherently heterogeneous disorder, and differences in age range, functioning level, and other demographic factors between studies may explain the complex pattern of findings. Moreover, all of these studies use the original or revised Autism Diagnostic Interview (ADI or ADI-R) to assess RRB. This instrument was not designed to separate out the different subcomponents of the RRB domain. Thus, contradictory findings may well relate to the use of a relatively gross summary metric of RRB symptom clusters, rather than a specific measure of RRB behavioral subcategories. A third possibility is that RRB does not have a simple anatomical correlate that can be revealed with brain-behavior correlations. Here, we will address these issues by using the Repetitive Behavior Scale-Revised (RBS-R), a behavioral questionnaire designed to assess subdomains of repetitive behavior, and by investigating the relationship of structural covariance among cortical and subcortical gray matter volume to specific RRB subdomains.

Structural covariance is a measure of morphological coupling among brain regions using MRI. Patterns of structural covariance have been interpreted as “networks” reflecting long time-scale developmental and plasticity effects [66]. These networks are not assumed to directly map onto synaptic connectivity, but instead reflect coordinated development over time. Though the genetic and microstructural basis of structural covariance is poorly understood, it has already found utility in describing reorganization of a changing brain during development (see [18] for review) and characterizing atypical brain structure in disorders like ASD. Zielinski et al. [64] examined the covariance structure of gray matter intensity related to the salience and default-mode networks in participants with ASD. They found that individuals with ASD showed a spatially restricted salience network, and more extensive default-mode network, in comparison to TD controls. Bernhardt et al. [6] also found reduced covariance of a structural network with a seed in dorsomedial prefrontal cortex in ASD compared to controls. Structural covariance has also been linked to particular behavioral measures in ASD; Bernhardt and colleagues found that covariance strength between frontal-insular cortex and occipital and temporoparietal cortices related to alexithymia (in both ASD and TD populations), and Sharda et al. [56] found that covariance strength of cortical thickness within a language network was modulated by verbal ability. Importantly, these studies use a seed-based approach where they evaluate the “integrity” or “extent” of a putative network based on a hub node that defines the network. While this method allows a detailed investigation of a predefined network, it limits exploratory power as structural relationships are not investigated more comprehensively. Additionally, group-based approaches limit the investigation of relationships between structural covariance and symptomatology that are specific to ASD. Along with these structural covariance abnormalities, ASD is increasingly characterized by functional connectivity (FC) abnormalities ([3, 15, 23]; see [61] for review) as well as aberrant neuroanatomical developmental trajectories (e.g., [62, 65]). Given these findings, we hypothesize that structural covariance is associated with individual differences in ASD symptomatology. In particular, we propose that structural covariance within the subcortex and between the subcortex and the cortex will relate to IS symptomatology in high-functioning ASD.

## Methods

Written informed consent was obtained from all adults and from the parents of participants under 18 years old; written assent was also obtained from all participants under 18 years old. Ethics approval for this study was granted by the NIH Combined Neuroscience Institutional Review Board under protocol number 10-M-0027.

### Participants

Fifty-five high-functioning males with an ASD (45 right handed, 1 left handed, 9 mixed) between 12 and 28 years old (18.39 ± 3.33) recruited from the Washington, DC metropolitan area participated in the study. Forty-eight participants met DSM-IV diagnostic criteria (26 Asperger’s syndrome, 19 high-function autism, 2 pervasive developmental disorder—not otherwise specified, and 1 with either Aspergers’s syndrome or high-functioning autism, which could not be determined due to lack of adequate developmental history) and seven participants met DSM-5 diagnostic criteria for ASD as assessed by an experienced clinician. All subjects received the autism diagnostic observation schedule (total *n* = 55; module 4, *n* = 46; module 3, *n* = 9, [43]), and most received the Autism Diagnostic Interview-Revised (ADI-R, *n* = 50, [44]) administered by a trained, research-reliable clinician. All ASD participants’ scores met cutoff for the category designated as “Broad ASD” according to criteria established by the NICHD/NIDCD Collaborative Programs for Excellence in Autism. Lainhart et al. [32] developed criteria that include an individual on the broad autism spectrum if s/he meets the ADI cutoff for autism in the social domain and at least one other domain, or meets the ADOS cutoff for the combined social and communication score. Exclusion criteria included an IQ <85 or any known co-morbid medical conditions, such as fragile X syndrome or other genetic disorder that could affect brain and behavioral development, and brain trauma/injury.

Full scale IQ scores were measured by the Wechsler Abbreviated Scale of Intelligence (WASI, *n* = 51), the Wechsler Adult Intelligence Scale-III (WAIS-III, *n* = 3), or the Wechsler Intelligence Scale for Children-IV (WISC-IV, *n* = 1).

### Repetitive behavior

Repetitive behavior severity was assessed with parent report on the RBS-R [33]. The RBS-R quantifies repetitive behavior in five behavioral subcategories: stereotyped, self-injury, compulsive, ritualistic, and IS. For analysis purposes, participants were split into more affected, high-scoring and less affected, low-scoring groups based on a median split of the raw scores in each of the five behavioral subcategories (Table 1). Subcategory scores were also analyzed as a continuous measure (see below).

**Table 1:** Demographic data and characteristics of sample

Variable	High-functioning ASD (*n* = 55)
Age (years), mean ± SD	18.39 ± 3.33
Full scale IQ (standard score), mean ± SD	111.96 ± 14.24
ADI social, mean ± SD (*n*)	19.98 ± 5.32 (50)
ADI communication, mean ± SD (*N*)	15.90 ± 4.53 (50)
ADI restricted/repetitive behaviors, mean ± SD (*n*)	5.50 ± 2.59 (50)
ADOS communication + social interaction, mean ± SD (*n*)	11.91 ± 4.10 (55)
Repetitive behavior (RBS-R raw scores)	
Insistence on sameness, mean ± SD	4.81 ± 5.21
Stereotyped behavior, mean ± SD	2.15 ± 2.26
Self-injury, mean ± SD	1.13 ± 1.31
Compulsive, mean ± SD	1.55 ± 1.96
Ritualistic, mean ± SD	2.89 ± 3.04

### Neuroimaging

One high-resolution T1-weighted structural image was obtained axially from each subject with a magnetization-prepared rapid gradient-echo (MPRAGE) array spatial sensitivity encoding technique (ASSET) sequence (124 slices, 1.2-mm slice thickness, 224 × 224 acquisition matrix, flip angle = 12°, field of view = 24 cm) on a 3T General Electric Signa Scanner (Milwaukee, WI) using an 8-channel head coil.

The FreeSurfer image analysis methods have been previously discussed in detail [10, 19, 20]. Briefly, FreeSurfer (version 5.1) was used to identify subcortical and cortical ROIs and calculate the average gray matter volume in each area. Seven bilateral subcortical ROIs were automatically segmented by FreeSurfer (thalamus, caudate, putamen, pallidum, hippocampus, amygdala, and accumbens), as well as 34 cortical ROIs per hemisphere based on a standard atlas [13]. While our planned analyses used averaged bilateral subcortical ROIs, we followed up by rerunning all subsequent analyses using 14 subcortical ROIs (the seven bilateral ROIs mentioned above separated by hemisphere). Figures from these analyses are included in the additional files (see Additional file 1: Figure S1, Additional file 2: Figure S2, and Additional file 3: Figure S3).

### Statistical analysis

Behavior was analyzed with the Kruskal-Wallis test, followed by the Wilcoxon rank sum test to ascertain if there were differences in the severity of RRB subcomponents as measured by the five behavior subcategories of the RBS-R in our ASD population.

Associations between IS symptoms and regional brain volume were assessed with Spearman’s rank correlation coefficients to guard against the influence of extreme and/or skewed IS scores, simultaneously covarying the nuisance effects of age, IQ, and intracranial volume (ICV) through partial correlation analyses. ICV, utilizing FreeSurfer’s automated ICV estimate that is equivalent for manual measurement (see [8]) was included in order to rule out variation in overall brain size from explaining the volume covariance results between pairs of regions. Multiple comparisons were corrected using the false-discovery rate (FDR) procedure [5].

To examine how structural covariance relates to IS, we defined two covariance metrics for analysis at either the group level (high vs. low IS, based on a median split) or continuously across all participants. For the median-split group comparison, structural covariance was defined as the Pearson’s product moment correlation of brain volumes between anatomical ROIs across subjects. Matrices of regional covariation were created for intra-subcortical, subcortico-cortical, and cortico-cortical relationships. Due to several ties at the median value of IS for the population, the median score was included in the group that resulted in the most even count in the high and low groups (low, 29 participants; high, 26 participants). In order to accommodate this difference in N-count for the low-high IS correlation comparisons, correlation values were transformed to population-level estimates prior to statistical comparison using the degrees of freedom, correcting for any bias due to the different numbers of subjects in each group [21, 47]. Group differences were then calculated between the groups’ adjusted Pearson’s r matrices. Significance was assessed using Monte Carlo simulation in the following manner. Each simulation shuffled group assignment into two new randomized groups such that each new group had an even mixture of original high and low IS participants. This simulation procedure reflects the null hypothesis that group assignment should not relate to anatomical covariation. An unrestricted randomization procedure would be overly conservative as the null distribution would frequently include samples identical or nearly identical to the actual experimental groups due to the relatively small sample sizes involved when splitting the entire sample in half. Covariation matrices were then created for each new group, and the differences were stored as the output of one simulation. Over 10,000 iterations, a distribution of correlation differences between the randomized groups was created for each Region X Region relationship and for the maximum correlation coefficient observed on any individual simulation. These distributions were used to compare the actual group differences resulting in, respectively, uncorrected, significant Region X Region differences, and significant Region X Region differences corrected for multiple comparisons.

Median-split approaches are known to suffer from statistical inefficiencies (e.g., [45]), and we therefore defined a more continuous analysis of how structural coupling relates to individual differences in IS severity. The measurement problem in this case is that each subject contributes only one data point from each anatomical region, and we seek to evaluate whether there is some covariation of the joint volume of pairs of regions that relates to IS, which is also a single measure for each subject. We addressed this measurement problem by essentially “deconstructing” a Pearson correlation coefficient across the group of subjects. A Pearson correlation coefficient (*r*) between two data series *X* and *Y* can be written as the normalized inner product of the standard scores (*z*-scores):

$$ r=\frac{1}{N-1}{\displaystyle \sum_{i=1}^N\left(\frac{X_i-\overline{X}}{s_X}\right)\left(\frac{Y_i-\overline{Y}}{s_Y}\right)=}\frac{1}{N-1}{\displaystyle \sum_{i=1}^N{z}_{X_i}{z}_{Y_i}} $$

where *N* is the length of the data series, $\overline{X}$ and $\overline{Y}$ are the mean of *X* and *Y*, and *s* is the sample standard deviation. In the continuous analysis for a pair of regions, we used the product of the *z*-scores that would be contributed by each individual subject (*i*) to an overall Pearson correlation as a measure of inter-regional association strength for that subject. This product was then correlated (Spearman) across subjects with each subject’s IS symptom score, partialling age, IQ, and ICV, allowing a form of the covariance of the volumes to be associated with IS in a continuous manner that should be similar in quality to the split-half version of the analysis. Multiple comparisons were corrected using the FDR procedure, determining the appropriate *p* value threshold needed for correction over all comparisons involving the subcortical brain regional volumes (including correlations between IS and single subcortical regional volumes, Region X Region covariation among subcortical region pairs, as well as between subcortical and cortical regions). The Spearman *rho*-values from this analysis indicated the magnitude of the statistical dependence between a specific Region X Region pair’s covariation and IS score, although one cannot directly interpret the positive/negative value of this measure in terms of joint volume increases or decreases. This analysis is referred to as the “Correlational Analysis” for the remainder of the paper.

Separate follow-up analyses were carried out for each of the four other subcategories defined in the RBS-R to ensure that our findings were most pronounced for IS. As indicated above, we also repeated the analysis with IS using 14 lateralized subcortical ROIs. Comparisons of structural correlates of IS in ASD versus TD controls was not possible, given that control participants would exhibit little or no variance in these behavioral measures (scores of 0). However, additional checks on the control of type I statistical errors in the correlation-based analyses were carried out in the additional files using permutation tests which confirmed the good performance of the FDR-correction procedure (see Additional file 4: Figure S4).

## Results

Consistent with previous reports, analysis of the RBS behavioral scores showed that IS symptoms were endorsed more often, and to a greater degree than any other RRB subcomponent, validating our particular interest in this behavior (*p* <.05). We first examined whether subcortical volumes correlated with IS scores. All seven subcortical volumes were negatively correlated with IS, though only the thalamus survived correction for multiple comparisons (*r* = −.39, *p* < .005; FDR *q* < .05). While correlations between cortical regional volumes and IS were not directly involved in our main hypotheses about the involvement of subcortical structures, they were carried out for purposes of comparison (see also Additional file 5). The vast majority of cortical ROIs had numerically negative correlations with IS, but only the right entorhinal cortex survived correction for multiple comparisons (*r* = −.47, *p* < .0005; FDR *q* < .05).

**Fig. 1:**
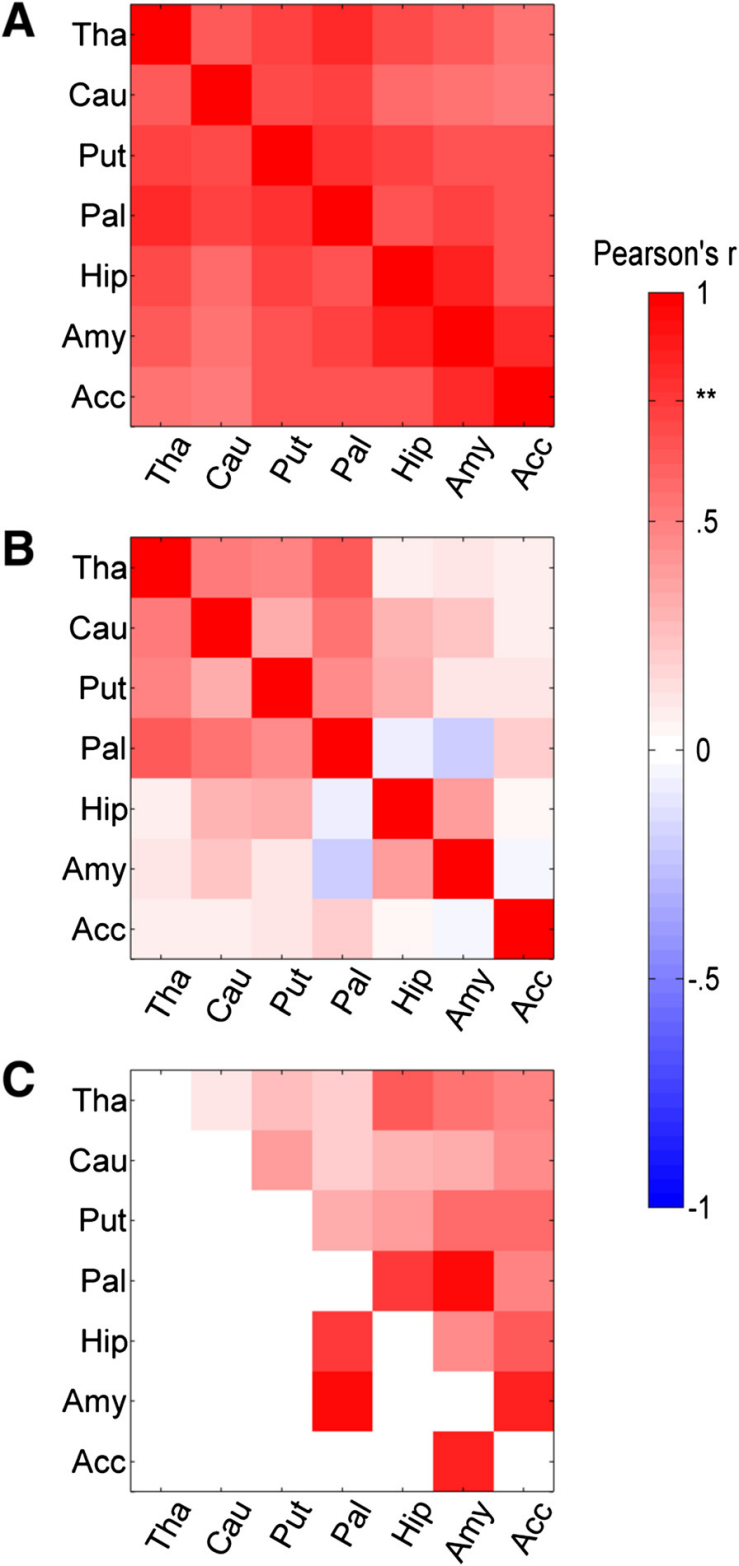
Intra-subcortical median-split analysis. Intra-subcortical volume Region X Region covariance matrices shown for **a** the high IS group, **b** low IS group, and **c** high-low difference. Color indicates the Pearson *r* value, or, in **c**, the r value difference. In **c**, the three differences surviving correction for multiple comparisons are displayed in the lower triangle. *Tha* thalamus, *Cau* caudate, *Put* putamen, *Pal* pallidum, *Hip* hippocampus, *Amy* amygdala, *Acc* nucleus accumbens

As described in the methods, two approaches were taken to assess structural covariance of subcortical structures and IS, a split-half group comparison and a continuous correlational approach. The relationship between IS and structural covariance was first assessed by a group comparison (between high and low IS groups). The high IS group had greater inter-regional correlations than the low IS group (high IS: *r* = .69 ± .09; low IS: *r* = .25 ± .24); out of 21 possible unique region pairs, three subcortical regional pairs survived correction for multiple comparisons (*p* < .05 via random permutation/Monte-Carlo): amygdala-pallidum, amygdala-accumbens, and hippocampus-pallidum (Fig. 1).

Similar to the subcortical inter-regional correlations, subcortico-cortical relationships were numerically greater in the high IS group relative to the low IS group for 27 % (131/476) of regional pairs (significant at *p* < .05, uncorrected), though none survived correction for multiple comparisons. Similarly, cortico-cortical analysis failed to yield results that survived correction for multiple comparisons.

**Fig. 2:**
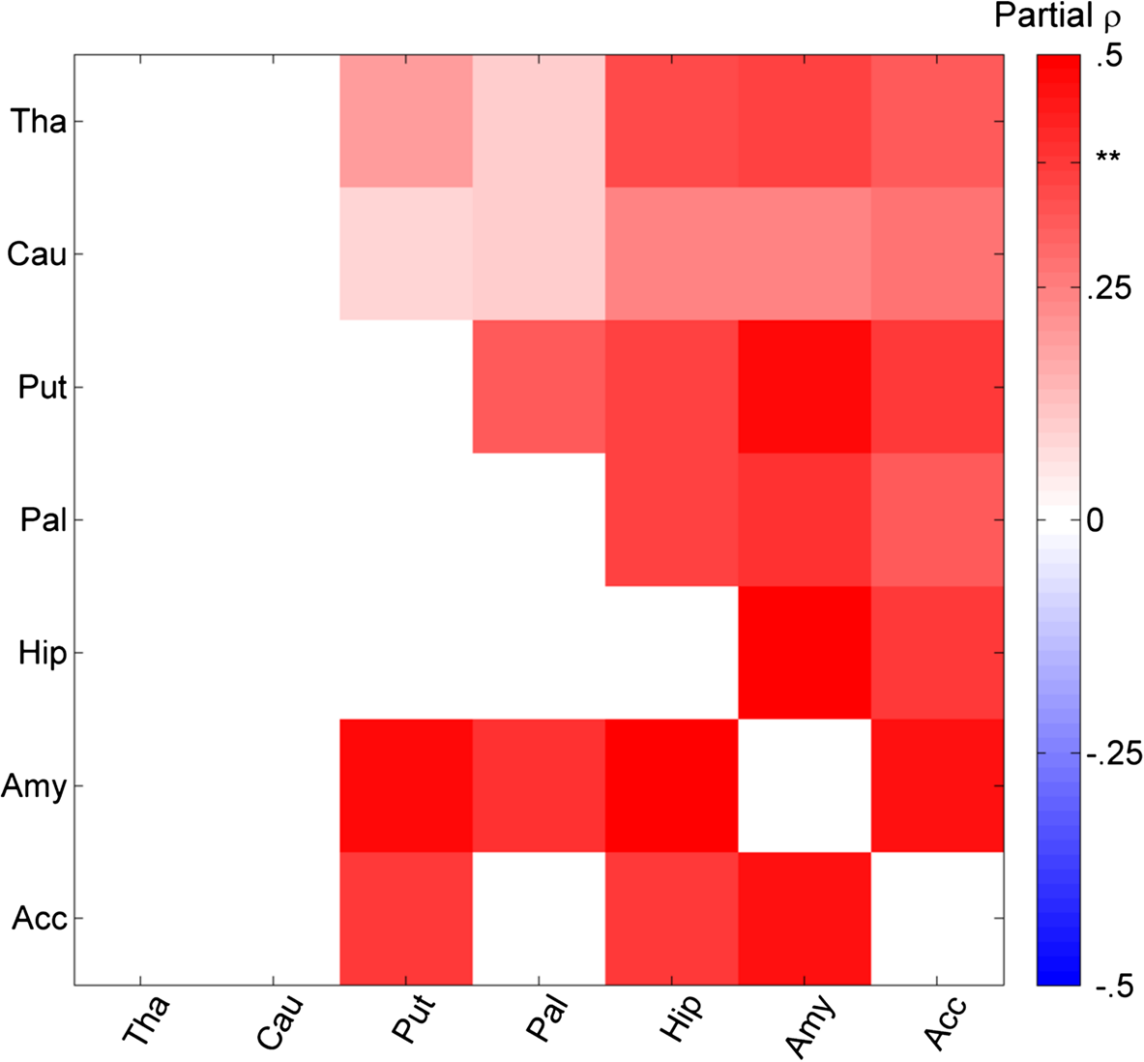
Intra-subcortical correlational analysis. Intra-subcortical correlational analysis showing the relationship between IS severity and Region X Region structural covariance, as measured by a single-subject covariance analog (see “Statistical analysis” section). FDR cutoff indicated by **. Relationships that survive FDR correction are displayed in the lower triangle. See Additional file 6: Table S1 for exact *r* and *p* values for significant relationships

The correlational analysis produced more robust results that were qualitatively consistent with the group comparisons described above (Fig. 2). In the subcortex, every Region X Region relationship was positively correlated with IS symptomatology (Spearman *r* = .29 ± .12), with six surviving correction for multiple comparisons (*p* < .0052 for all; FDR, *q* < .05; shown in the lower triangle in Fig. 2 with labels shown in Fig. 1; see Additional file 6: Table S1 for a complete list of region pairs that survived correction and Additional file 7: Figure S5 for example scatter plots of the relationship between IS and Region X Region association strength). Most of the subcortex was implicated in this analysis including the putamen, amygdala, hippocampus, pallidum, and accumbens.

**Fig. 3:**
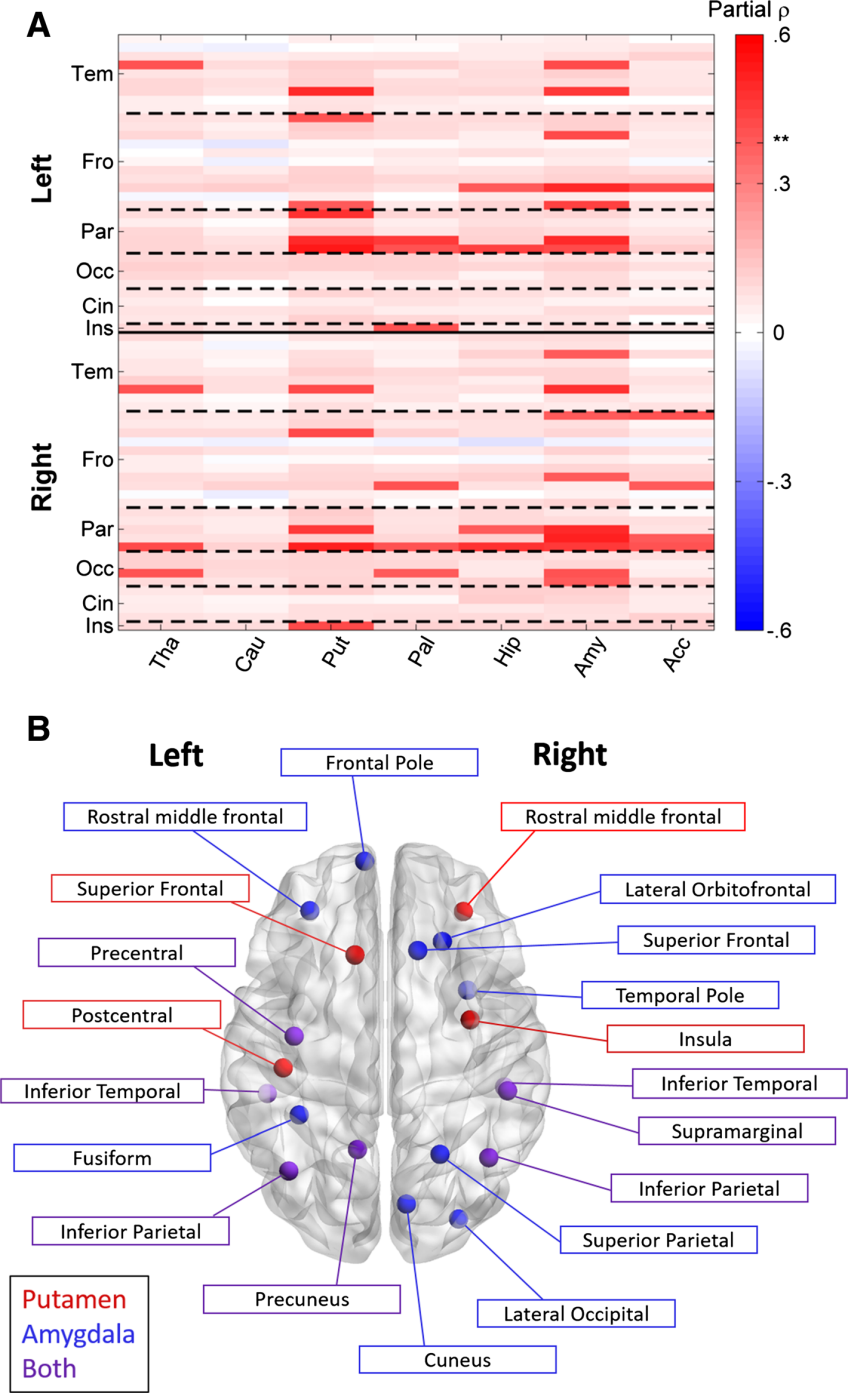
Cortico-subcortical correlational analysis. Subcortical-cortico correlational analysis showing the relationship between IS severity and Region X Region structural covariance (**a**), as measured by a single-subject covariance analog (see “Statistical analysis” section). Cortical regions are ordered by hemisphere ^1^ and FDR cutoff is indicated by **. Relationships that survive FDR correction are highlighted by making non-significant differences partially transparent. See Additional file 8: Table S2 for exact *r* and *p* values for significant relationships. **b** shows a subset of these significant relationships: cortical regions whose relationship with the putamen (*red*), amygdala (*blue*), or both (*purple*) significantly related to IS severity

Subcortico-cortical relationships showed the same general trend as the group comparison with the covariance of 41 pairings positively associated with IS symptoms surviving correction for multiple comparisons (*p* < .0052 for all; FDR, *q* < .05; shown in bright colors in Fig. 3 with labels included in legend). The putamen and amygdala each participated in at least ten of these relationships (see Additional file 8: Table S2 for a complete list of region pairs surviving correction). Finally, although it was not directly related to our hypothesis about subcortical structural covariation and IS, we examined cortico-cortical covariation in an analogous manner for comparison (see Additional file 5). No ROI pairs survived correction for multiple comparisons in the continuous analysis of cortico-cortical covariation.

The preceding analyses using regional volume and volume covariance were repeated for the other four behavioral subcategories of the RBS-R for comparison (stereotyped, self-injurious, compulsive, and ritualistic behaviors). The volume of the right precuneus was negatively correlated with ritualistic behavior across subjects (*r* = −.46, *p* < .0005). For the RBS subcategories of stereotyped, self-injurious, and compulsive behaviors, there were no results that survived statistical correction for multiple comparisons. Furthermore, none of the volume covariance analyses yielded corrected results for the non-IS behavioral subcategories.

## Discussion

This study found that increased structural covariance of gray matter volume relates to IS severity in adolescents and adults with high-functioning ASD. No other behavioral subcategory of RRB was found to be significantly related to anatomical covariance between structures. The fact that our neuroanatomical findings were most pronounced for IS is perhaps unsurprising for this high-functioning ASD sample, given that IS symptomatology was the most endorsed RRB symptom. ASD is a highly heterogeneous disorder, and understanding neuroanatomical correlates in terms of behavioral dimensions rather than the disorder as a whole is an important step to consolidating discordant results in the literature [40]. This study provides evidence that the range of behaviors that comprise RRB may be too broad to consistently map onto neuroanatomy, thus requiring more selective behavioral distinctions to fully identify subtle brain-behavior relationships. Moreover, using the traditional approach of examining correlations between RRB and subcortical volumes revealed modest to weak relationships. Instead, the association between IS and structural covariance of the subcortex (both within subcortex and subcortical-cortical) was more robust. This points to the potential value of utilizing structural covariance as a neuroanatomical marker in ASD.

Our findings implicate subcortical structures as related to IS including parts of the striatum and amygdala. In both the group comparison and correlational analysis, more severe IS symptoms were related to increased covariation among all structures. While both analyses were qualitatively similar, the median-split reduced statistical power and partially masked the extent of this relationship, serving as a clear example as to why statisticians recommend against the use of median splits when a more continuous correlation/regression analysis is possible (e.g., [45]). The correlational analysis revealed effects across the majority of the subcortex and many cortical-subcortical relationships. These findings raise the possibility of a widespread, atypical growth factor overriding normal developmental variation in subcortical structural volume associated with IS, perhaps reducing the relative independence, or modularity, of structural “networks.” IS’s genetic specificity [9, 34, 55, 58] allows for this possibility, though longitudinal studies are needed to adequately pursue this idea. Moreover, given that (gray matter) structural coupling is related to, but independent of, physiological coupling (functional connectivity, or FC) [1], these relationships may reflect aberrant functional networks underlying variation in IS. Future work marrying these structural covariance approaches with functional neuroimaging methods (e.g., FC) is needed to investigate this possibility.

We did not find pervasive, robust relationships between striatal volume and RRB symptoms. Previous work has failed to find consistent brain-behavior relationships between RRB and neuroanatomy, even while consistent neuroanatomical differences between ASD and TD groups are reported. For non-IS RRB, our null result may be due to the small variance in our population’s behavioral scores, rather than indicating a true lack of anatomical correlates. Indeed, besides IS, our one brain-behavior relationship involved ritualistic behavior, the second most strongly endorsed behavior in our population. This implies that relating other behaviors to structure may be possible in a sample with differing RRB characteristics. IS, however, did have a large range of severity scores in this sample, and so this explanation of our null result is unsatisfactory. Another possibility may be that IS does not have a consistent relationship with individual structures but should instead be understood in terms of abnormal structural covariance among a large number of interacting regions. In general, these findings help to explain prior discordant work on the relationship between abnormal subcortical volume in ASD and RRB. Failing to explicitly separate IS from other RRB may lead to obscuring brain-behavior relationships that are inconsistent across RRB subcomponents, or worse, corruption of the underlying relationship between neuroanatomy and RRB. By identifying subcomponents of RRB symptoms, and using structural covariance, this study identifies many brain regions outside the few traditionally studied as important for IS.

Among the regions identified, the amygdala was particularly prominent, participating in both subcortical and cortical connections related to IS. Specifically, correlational analysis indicated that relationships between the amygdala and bilateral temporal and parietal regions, left frontal, right occipital, and every subcortical region except the caudate were related to IS. This central role for the amygdala in IS is not without precedent. A study by Lingawi and Balleine [41] has shown that the amygdala interacts with the striatum during habit formation and that disrupting this connection affects habit acquisition. Another work has hypothesized that the amygdala may be divorced from its normal involvement in social and emotional understanding in individuals with ASD, and instead mediate the imposition of routines [16]. Our data shows that anatomical associations between the amygdala and basal ganglia, as well as parts of the cortex, relate to IS in a manner supportive of that claim. Alternatively, IS, unlike lower-level repetitive sensory and motor behaviors, has been linked with anxiety in ASD [51]. The amygdala has long been implicated in anxiety disorders [11], and structural and functional connectivity involving the amygdala has been related to anxiety in many fMRI studies (see [31] for a review). IS’s relationship to anxiety in ASD makes it qualitatively different from other RRB and would explain the amygdala’s selective involvement. Of course, these explanations are not mutually exclusive, as the same neurocircuitry that underlies anxiety may mediate the imposition of routines in ASD.

The striatum was also implicated in our covariance analyses. We identified a relationship between IS and putamen covariance, both with the rest of the subcortex and the cortex, particularly parietal areas. Work by Padmanabhan et al. [48] reported a similar increase in functional connectivity between the striatum and parietal areas in ASD versus controls, though they did not report any behavioral correlations. However, we did not find any relationship between the caudate and IS symptomatology. Caudate volume has previously been linked with IS [35, 38], in contrast to our null result. This discrepancy in findings could be attributable to numerous factors including methodology (e.g., hand tracings and voxel-based morphometry in the former studies versus semi-automated surface-based methods used here) or demographic characteristics (e.g., older subjects with ASD in the current study). Given the dynamic nature of subcortical brain structure development, including the caudate nucleus, it is feasible that the associations with behavior (including IS) will change depending on the developmental window examined.

More broadly, our results indicate that IS is related to a general increase in structural covariation among the majority of subcortical regions. This reaffirms the general abnormal development of these structures in ASD, but it is important to note that this study showed that increased coupling was related only to IS, rather than the disorder as a whole. On this point, it is interesting to note that Di Martino et al. [14] found increased physiological coupling (FC) among many subcortical structures in ASD compared to controls. They also found that RRB within the ASD group correlated with right putamen-superior temporal gyrus FC, though the behavioral correlation did not survive correction for multiple comparisons. More recently, Delmonte et al. [12] found that increased FC between middle frontal gyrus and striatum (caudate nucleus) was associated with RRB in ASD. While the relationship between anatomical and physiological covariation is likely to be complex [1, 29], these physiological results taken together with our current anatomical results highlight an important role for subcortical structures in the pathophysiology of ASD more generally, and of RRB and IS in particular.

### Limitations and future directions

The current study has a number of limitations. Similar to all studies of ASD, the generalizability of our work is related to the characteristics of the sample. The findings presented here may not represent what would be found in a sample of individuals with both ASD and an intellectual disability. Indeed, the very fact that IS was the most frequently endorsed RRB is testament to how different our sample may be from a sample with, say, more severe repetitive sensory and motor behaviors. Considering that prior evidence suggests that RRB largely breaks down into two subcomponents, one could hypothesize that similar work on a population with a large variability of repetitive sensory and motor behaviors may uncover a different dimension of structural association. Sex may also play a role, as it has previously been shown that boys with ASD display higher rates of RRB compared to girls with ASD [25], though this effect was not seen in high-functioning ASD [46]. Nevertheless, the specific relationship between anatomical covariance and RRB may interact with sex, which we are unable to explore in the current study.

This work is also restricted by its reliance on an anatomical atlas. Though a helpful starting point, segmenting the cortex a priori will inevitably lead to underspecifying the true brain-behavior relationships. As a first step, using an atlas allows an agnostic inquiry of brain-behavior relationships by reducing the number of areas examined, thus increasing statistical power; but follow-up studies are necessary to better define regional specificity. It should also be noted that in this study, our cortical analyses may have been underpowered given the large number of areas we evaluated (particularly vis-à-vis the smaller number of subcortical regions). Thus, we certainly cannot rule out the involvement of cortico-cortical covariance in RRB. Moreover, recent work supporting cortical involvement of behavioral flexibility has shown increased frontal activation during a task-set switching paradigm in ASD compared to controls, even while behavioral performance is equivalent, suggesting less efficiency when switching behaviors [63]. This lowered switching efficiency may relate to work by Uddin et al. [60], who have shown that task-evoked networks are less discriminable from intrinsic networks in ASD versus controls, and the level of discriminability inversely relates to RRB. Though causal direction is unclear, this work provides compelling evidence that the flexibility of cortical neural organization mirrors behavioral flexibility, and future work may outline anatomical correlates of these functional characteristics.

In a similar vein, gray matter volume is but one gross metric of anatomy and will limit the kinds of brain-behavior relationships we can uncover. Further work could extend the structural metrics used to gray matter thickness, surface area, curvature, and subcortical shape. Here, to remain as parsimonious as possible, we restricted our analysis to gray matter volume, which is a consistent measure in both the cortex and subcortex.

## Conclusions

We identified neuroanatomical correlates of IS in high-functioning ASD. Unlike previous studies, we did not find that cortical volume directly related to symptomatology; rather, morphological coupling as measured by structural covariance was identified as behaviorally relevant. Specifically, increased covariation among most of the subcortex and some cortical regions was related to IS severity. This widespread, robust structural correlate of IS provides a neuroanatomical basis for IS’s characterization as a separate behavioral dimension of RRB in ASD, and helps further specify neurobiological underpinnings of RRB in general.

## Endnotes

^1^ Ordering of hemisphere regions from top to bottom (equivalent for both hemispheres): (Tem)—entorhinal cortex, parahippocampal gyrus, temporal pole, fusiform gyrus, superior temporal gyrus, middle temporal gyrus, inferior temporal gyrus, tranverse temporal cortex, banks of the superior temporal sulcus; (Fro)—superior frontal gyrus, caudal middle frontal gyrus, rostral middle frontal gyrus, pars opercularis, pars orbitalis, pars triangularis, medial orbitofrontal cortex, lateral orbitofrontal cortex, frontal pole, paracentral gyrus, precentral gyrus; (Par)—postcentral gyrus, supramarginal gyrus, superior parietal cortex, inferior parietal cortex, preceuneus cortex; (Occ)—lingual gyrus, pericalcarine cortex, cuneus cortex, lateral occipital cortex; (Cin)—rostral anterior/caudal anterior/posterior/isthmus divisions, corpus callosum; (Ins)—insula

## Additional files

Additional file 1: Figure S1. Intra-Subcortical Median-Split Analysis. Intra-Subcortical volume Region X Region covariance matrices with separate subcortical ROIs for each hemisphere shown for (A) the High IS group, (B) Low IS group, and (C) High-Low difference. Color indicates the Pearson *r* value, or, in C, the r value difference. In C, the two differences surviving correction for multiple comparisons are displayed in the lower triangle. Tha = Thalamus, Cau = Caudate, Put = Putamen, Pal = Pallidum, Hip = Hippocampus, Amy = Amygdala, Acc = Nucleus Accumbens. (PNG 367 KB) Additional file 2: Figure S2. Intra-Subcortical Correlational Analysis. Intra-Subcortical correlational analysis with separate subcortical ROIs for each hemisphere showing the relationship between IS severity and Region X Region structural covariance, as measured by a single-subject covariance analog (see “Statistical analysis” section). FDR cutoff indicated by **. Relationships that survive FDR correction are displayed in the lower triangle. (PNG 54.0 KB) Additional file 3: Figure S3. Cortico-Subcortical Correlational Analysis. Subcortical-cortico correlational analysis with separate subcortical ROIs for each hemisphere showing the relationship between IS severity and Region X Region structural covariance, as measured by a single-subject covariance analog (see “Statistical analysis” section). Cortical regions are ordered by hemisphere ^1^ and FDR cutoff is indicated by **. Relationships that survive FDR correction are highlighted by making non-significant differences partially transparent. (PNG 70.9 KB) Additional file 4: Figure S4. Permutation Test Control. The number of regional combinations detected across 5000 random iterations is shown as a frequency histogram, with number of ROI-ROI combinations shown on the x-axis and number of random iterations shown on the y-axis. The number of ROI-ROI combinations detected in the actual data (53/504 tests) is highly significant (*P* < .0007) and shown as a vertical dashed line for reference. (PNG 395 KB) Additional file 5:
**Supplemental Analyses.** (DOCX 27.1 KB) Additional file 6: Table S1. Subcortical-Subcortical Region X Region Pairs with Significant (corrected) Correlations with IS. (XLSX 45.6 KB) Additional file 7: Figure S5. Correlational Analysis Scatter Plots. Example scatter plots of three Region X Region relationships that significantly correlated with IS. Region X Region association strength is calculated as the product of an individual’s regional volumes transformed to z-scores (see “Methods” section in main text). (PNG)144 KB) Additional file 8: Table S2. Subcortical-Cortical Region X Region Pairs with Significant (corrected) Correlations with IS. (XLSX 88.0 KB)

## Abbreviations

ADI: autism diagnostic interview
ADI-R: Autism Diagnostic Interview-Revised
ASD: autism spectrum disorder
DSM-IV: the diagnostic and statistical manual of mental disorders IV
FC: functional connectivity
FDR: false-discovery rate
fMRI: functional magnetic resonance imaging
ICV: intracranial volume
IS: insistence on sameness
MRI: magnetic resonance imaging
RBS-R: Repetitive Behavior Scale-Revised
RRB: restricted and repetitive behavior
TD: typically developing
