# Supplementary figures and images for: Insistence on sameness relates to increased covariance of gray matter structure in autism spectrum disorder
Source: Mol Autism. 2015 Oct 1;6:54. doi: 10.1186/s13229-015-0047-7 (PMC4591718; doi:10.1186/s13229-015-0047-7)

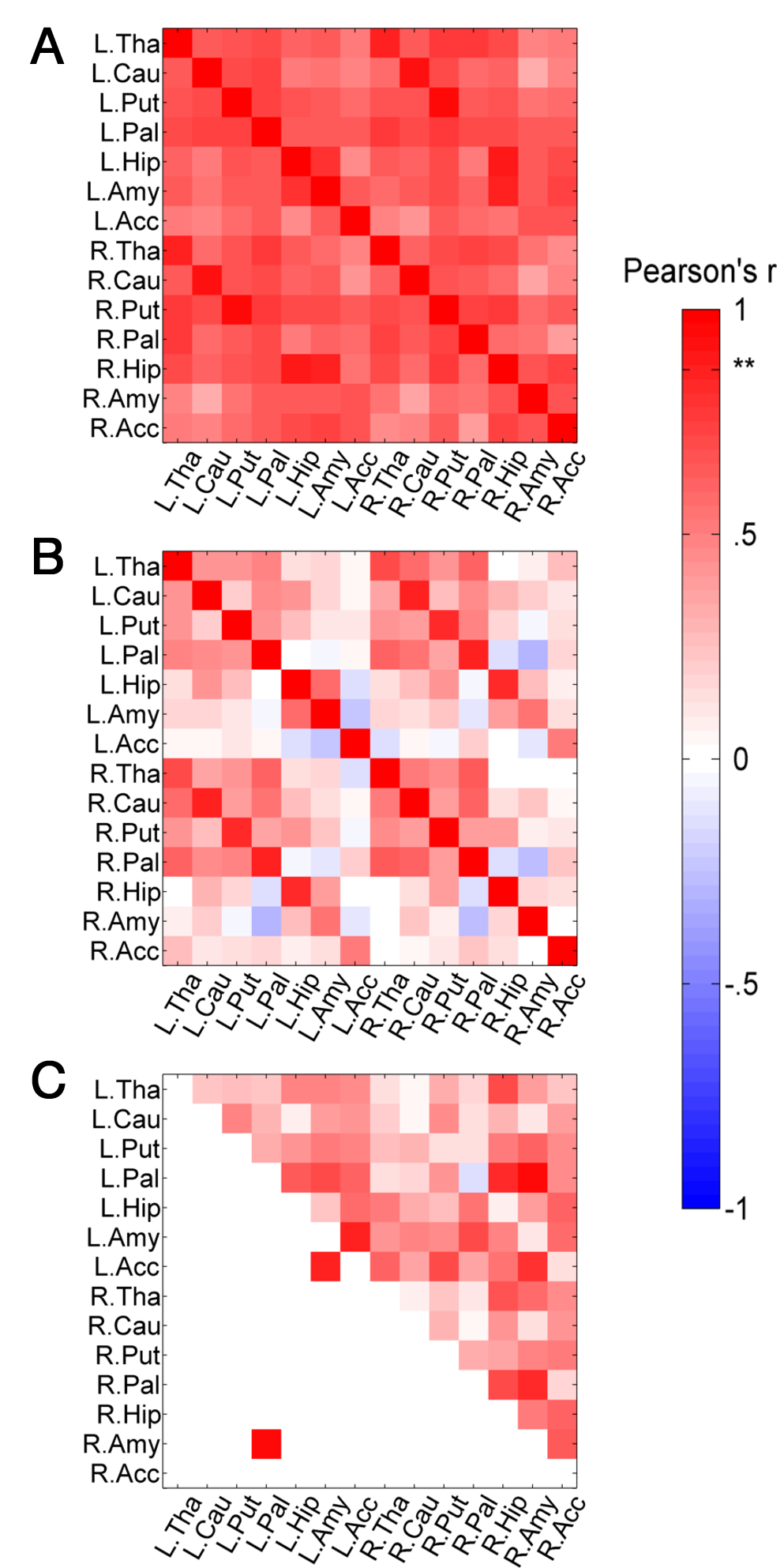

Supplement: Additional file 1: Figure S1. — Intra-Subcortical Median-Split Analysis. Intra-Subcortical volume Region X Region covariance matrices with separate subcortical ROIs for each hemisphere shown for (A) the High IS group, (B) Low IS group, and (C) High-Low difference. Color indicates the Pearson r value, or, in C, the r value difference. In C, the two differences surviving correction for multiple comparisons are displayed in the lower triangle. Tha = Thalamus, Cau = Caudate, Put = Putamen, Pal = Pallidum, Hip = Hippocampus, Amy = Amygdala, Acc = Nucleus Accumbens. (PNG 367 KB) [file 13229_2015_47_MOESM1_ESM.png]

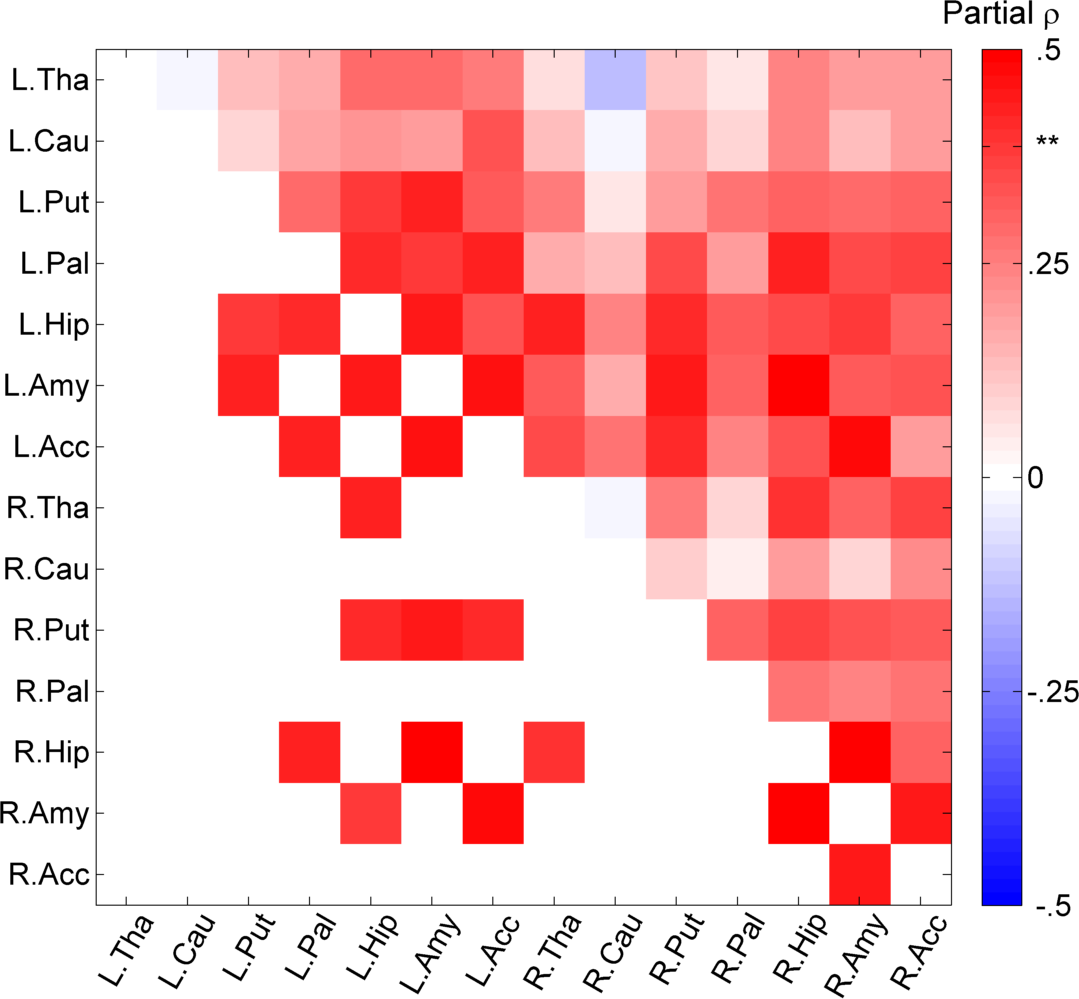

Supplement: Additional file 2: Figure S2. — Intra-Subcortical Correlational Analysis. Intra-Subcortical correlational analysis with separate subcortical ROIs for each hemisphere showing the relationship between IS severity and Region X Region structural covariance, as measured by a single-subject covariance analog (see “Statistical analysis” section). FDR cutoff indicated by **. Relationships that survive FDR correction are displayed in the lower triangle. (PNG 54.0 KB) [file 13229_2015_47_MOESM2_ESM.png]

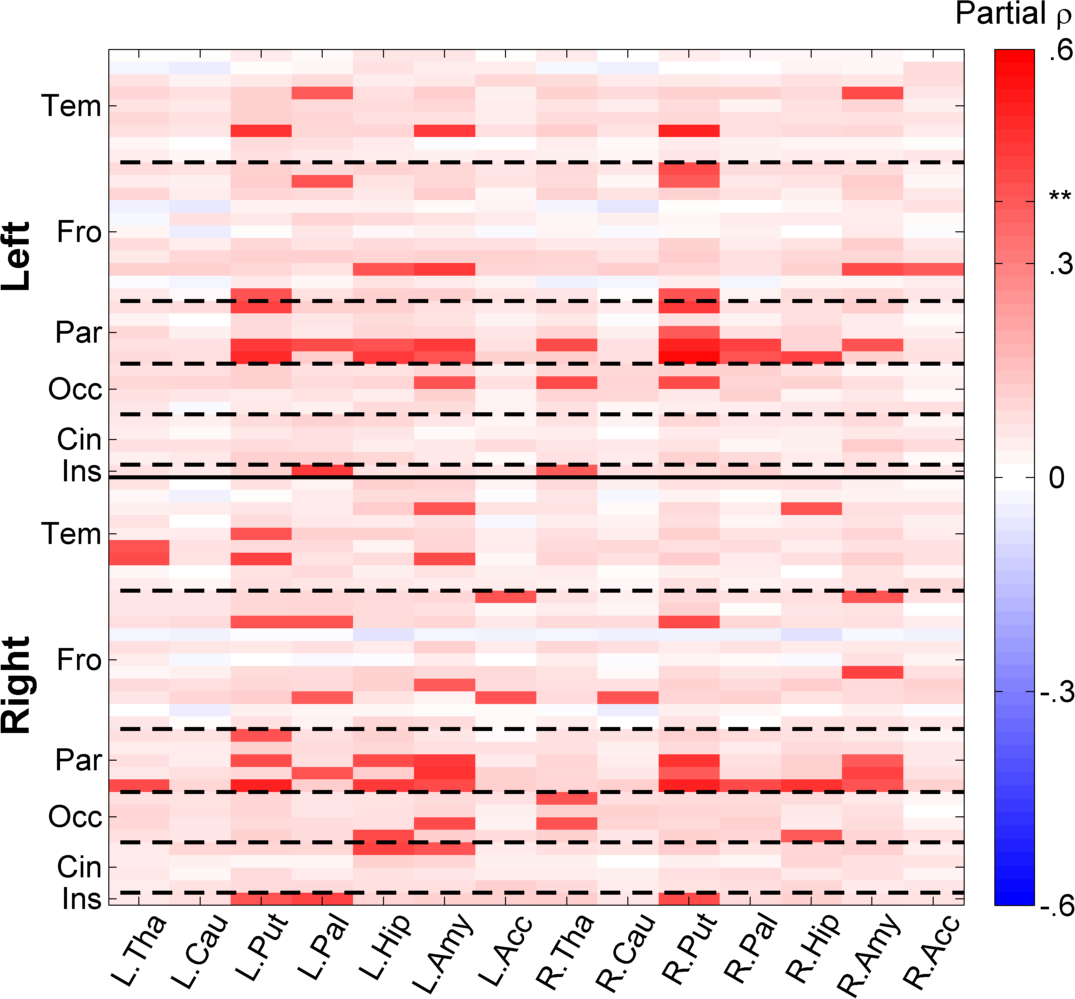

Supplement: Additional file 3: Figure S3. — Cortico-Subcortical Correlational Analysis. Subcortical-cortico correlational analysis with separate subcortical ROIs for each hemisphere showing the relationship between IS severity and Region X Region structural covariance, as measured by a single-subject covariance analog (see “Statistical analysis” section). Cortical regions are ordered by hemisphere 1 and FDR cutoff is indicated by **. Relationships that survive FDR correction are highlighted by making non-significant differences partially transparent. (PNG 70.9 KB) [file 13229_2015_47_MOESM3_ESM.png]

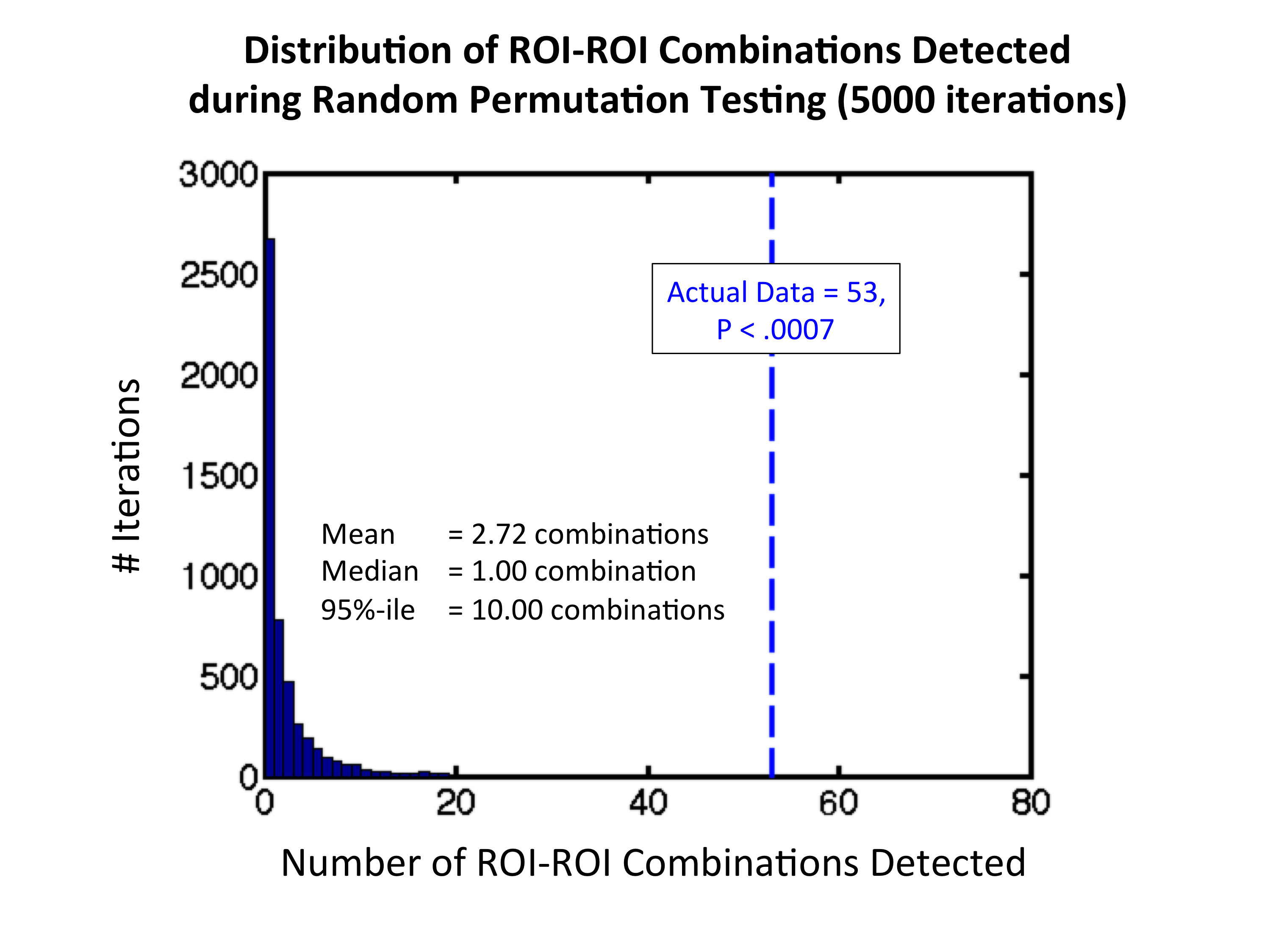

Supplement: Additional file 4: Figure S4. — Permutation Test Control. The number of regional combinations detected across 5000 random iterations is shown as a frequency histogram, with number of ROI-ROI combinations shown on the x-axis and number of random iterations shown on the y-axis. The number of ROI-ROI combinations detected in the actual data (53/504 tests) is highly significant (P < .0007) and shown as a vertical dashed line for reference. (PNG 395 KB) [file 13229_2015_47_MOESM4_ESM.png]

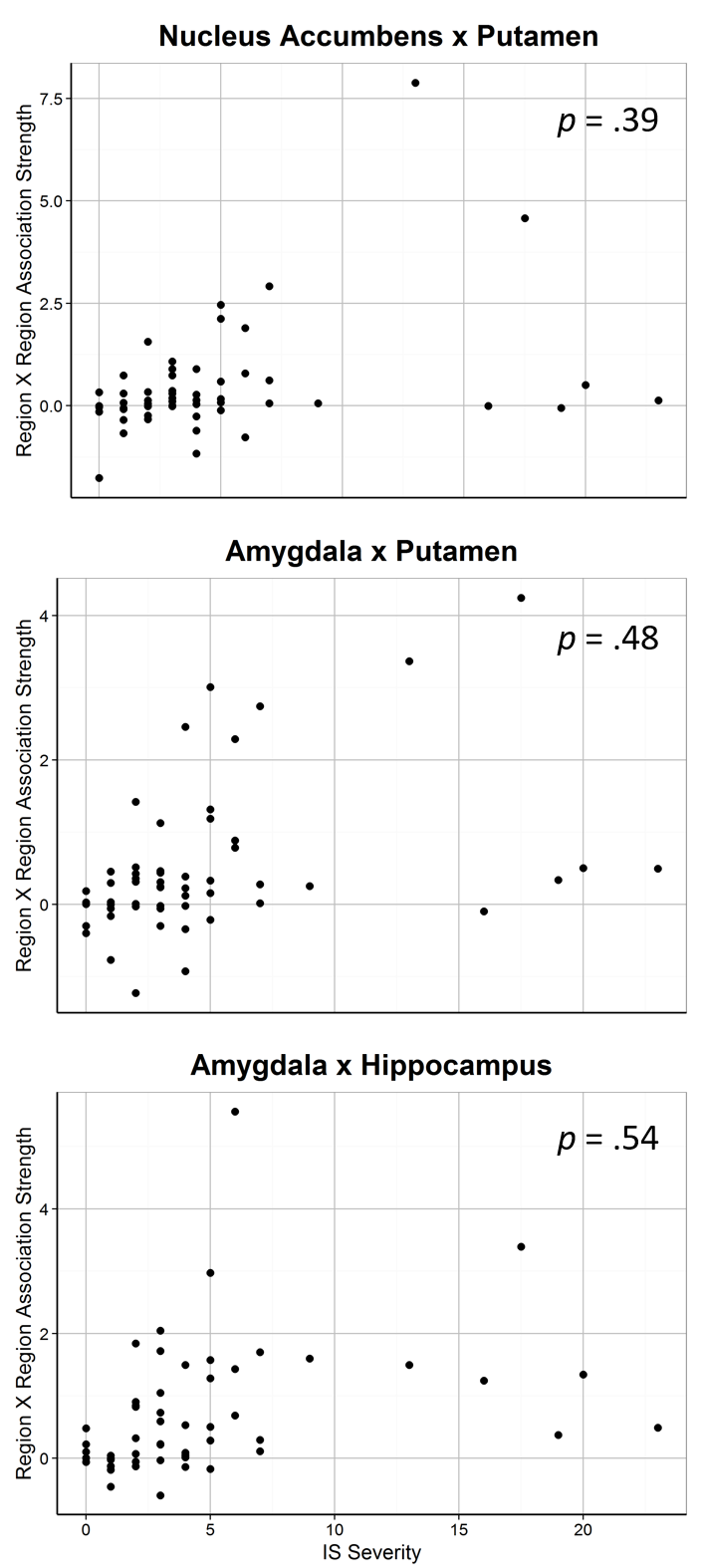

Supplement: Additional file 7: Figure S5. — Correlational Analysis Scatter Plots. Example scatter plots of three Region X Region relationships that significantly correlated with IS. Region X Region association strength is calculated as the product of an individual’s regional volumes transformed to z-scores (see “Methods” section in main text). (PNG)144 KB) [file 13229_2015_47_MOESM7_ESM.png]
