# Supplementary material for: Insistence on sameness relates to increased covariance of gray matter structure in autism spectrum disorder
Source: Mol Autism. 2015 Oct 1;6:54. doi: 10.1186/s13229-015-0047-7 (PMC4591718; doi:10.1186/s13229-015-0047-7)
Supplement: Additional file 5: — Supplemental Analyses. (DOCX 27.1 KB) [file 13229_2015_47_MOESM5_ESM.docx]

**Supplemental Analyses**

**Permutation Test Control Analysis**

In the continuous correlational analyses with Insistence on Sameness (IS) in the main text, it was not possible to compare directly to the same measures taken for control participants, since IS measures would rarely be non-zero. In order to further check the performance of our False Discovery Rate correction for Type I statistical errors when examining the full family of tests conducted (N=504 comparisons for subcortical single volume correlations with IS, subcortico-subcortical correlations, and subcortico-cortical correlations), we conducted a set of permutation tests in which we randomly shuffled the IS scores across the 55 ASD participants and re-calculated all of the tests (5000 random iterations). All nuisance covariates (Age, IQ, and ICV) remained with the original participants' regional volume data. On each iteration, the number of tests surviving the original FDR-corrected *p*-value (*p*<.0052) were recorded and then tabulated across iterations to determine the corrected *p*-value of the actual results (simply the percentile rank of the actual results detected, 53/504 tests, relative to the permuted results).

The results of this control analysis are shown in Supplemental Figure 4. The mean number of tests surviving *p*<.0052 across iterations was 2.72/504 (median = 1.0/504; 95%-ile = 10/504). If one compares the average number of random results to the number detected in the actual data, we find that 2.72/53 = .0513, which is very close to what should be detected when controlling FDR to q<.05 (i.e. approximately 5% or less of the detected results are expected to be spurious). When calculating the overall Type-I error likelihood of the total number of detected results in the actual data (53/504) using the permuted data, we estimate that the likelihood of detecting 53 results or more by chance is *p*<.0007. It appears then that the FDR-correction procedure is behaving appropriately, and it is very unlikely that our results are explained by chance.

**Relative Strength of Results involving Subcortical versus Cortical Regions**

In the Background section of the main text, we articulated the *a priori* reasons for examining structural covariation of subcortical structures with IS. However, it is reasonable to ask whether the results involving subcortical regions are actually more prevalent than those involving cortical regions only. Accordingly, we applied the same *p*-value threshold used in the main results to correlational analyses of single cortical volumes with IS (68 tests) and cortico-cortical structural-covariation analyses (68*67/2 = 2278 tests), for a total of 2346 tests. When comparing to the results for subcortex alone (7 single-volume and 21 subcortico-subcortical tests or 28 total tests), we found that 7/28 tests were detected at *p*<.0052 for a rate of 25%. The rate detected for subcortico-cortical tests was 46/476, or 9.66%. In contrast, the corresponding analyses for cortical regions detected 88/2346 results, or 3.75%. In terms of the raw rates of detection, it appears then that results involving subcortical regions (53/504 = 10.52%) are indeed more prevalent than those only involving cortex (88/2346 = 3.75%).

While these ratios are in line numerically with the hypothesis that structural covariance relationships involving subcortex are more associated with IS than those involving cortex alone, it is difficult to compare these rates statistically. A Chi-Square test of the ratios involving subcortex (53/504) versus cortex only (88/2346) is highly significant (χ^2^ df1 = 38.95, P<.0001), although the independence assumption for individual observations that underlies this test may be violated in this case. An alternative is to perform bootstrap resampling, randomly selecting subjects with replacement to construct many bootstrapped samples (each with N=55 randomly selected ASD participants), allowing us to derive variability estimates of the subcortical and cortical rates and compare them statistically.

In 1000 bootstrapped samples using a less restrictive *p*-value of *p*<.05 on individual tests (in order to prevent many iterations with 0 results for either subcortex or cortex), we find that the mean detection rate for subcortical tests is 42.43% (SD = 13.54%) and for cortical tests is 27.47% (SD = 9.33%). Out of the 1000 bootstrapped samples, the subcortical detection rate was greater than the cortical detection rate in 96.8% of samples, for a 2-tailed *p*-value of *p*<.064 (a statistical trend). Taken together, these results provide some tentative support for greater involvement of subcortical structures in IS behaviors for high-functioning ASD participants.
